# Clinical Outcomes and Left Ventricular Functional Remodeling after Extracorporeal Membrane Oxygenation Assisted Percutaneous Coronary Intervention in Patients with Ischemic Cardiomyopathy: A Single-Center Retrospective Observational Study of 76 Cases

**DOI:** 10.31083/j.rcm2509317

**Published:** 2024-09-06

**Authors:** Yi Dong, Zheng Xu, Xiao-fu Dai, Liang-wan Chen, Zhi-qin Lin

**Affiliations:** ^1^Department of Cardiovascular Surgery, Fujian Heart Medical Center, Fujian Medical University Union Hospital, 350001 Fuzhou, Fujian, China; ^2^Key Laboratory of Cardio-Thoracic Surgery, Fujian Medical University, Fujian Province University, 350001 Fuzhou, Fujian, China

**Keywords:** extracorporeal membrane oxygenation, percutaneous coronary intervention, ischemic cardiomyopathy

## Abstract

**Background:**

Ischemic cardiomyopathy (ICM) is a common condition that leads to left ventricular (LV) functional remodeling and poor prognosis. Extracorporeal membrane oxygenation (ECMO) can provide temporary circulatory support and facilitate percutaneous coronary intervention (PCI) in patients with ICM and hemodynamic instability. However, the impact of ECMO-assisted PCI on LV functional remodeling and clinical outcomes in ICM patients is unclear.

**Methods:**

We retrospectively analyzed 76 patients with ICM who underwent ECMO-assisted PCI at our institution between January 2013 and December 2022. We assessed the changes in LV functional remodeling using echocardiography at baseline and 12 months after the procedure. We also evaluated the incidence of major adverse cardiac and cerebrovascular events (MACCEs) and ECMO-related complications during hospitalization and at one-year follow-up.

**Results:**

The mean baseline left ventricular ejection fraction (LVEF) was 29.98 ± 2.65%. The rate of complete revascularization was 58%. The median duration of ECMO support was 38.99 hours. The most common ECMO-related complications were bleeding (8%) and lower extremity ischemia (5%). The one-year mortality rate was 30%. The overall freedom from MACCEs at 12 months was 59% (95% confidence interval (CI): 49–71%). LVEF increased significantly after the procedure from baseline to 6 months, yet decreased slightly at 12 months, although it was still higher than the baseline value. Wall motion score index (WMSI), end-diastolic volume index (EDVI), and end-systolic volume index (ESVI) decreased significantly from baseline to 12 months, indicating an improvement in LV function and a reduction in LV size.

**Conclusions:**

In a high-volume tertiary center with extensive experience in advanced heart failure therapies and a dedicated ECMO team, ECMO-assisted PCI demonstrated feasibility and safety in patients with ischemic cardiomyopathy. However, the rate of complete revascularization was modest at 58%. Despite the high-risk profile of the patients, ECMO-assisted PCI was associated with a significant improvement in LV functional remodeling and a favorable 12-month survival rate. Further prospective studies are needed to confirm these findings and to identify the optimal patient and device selection criteria for ECMO-assisted PCI.

## 1. Introduction

Ischemic cardiomyopathy (ICM), a prevalent condition affecting millions worldwide, is characterized by a progressive decline in left ventricular (LV) function due to coronary artery disease [1]. While the term ‘cardiomyopathy’ traditionally refers to primary myocardial disorders not associated with coronary artery disease, ‘ischemic cardiomyopathy’ is used here to describe systolic dysfunction resulting from ischemic damage, leading to what is clinically categorized as heart failure. The multifaceted risk factors for ICM include modifiable elements such as hypertension, diabetes, and lifestyle factors, as well as non-modifiable factors such as age and genetic predisposition [2, 3]. The pathophysiological hallmark of ICM is LV functional remodeling, a complex process involving changes in the size, shape, and function of the heart due to ongoing ischemia. This remodeling can lead to heart failure, arrhythmias, and other severe cardiovascular complications, further exacerbating the morbidity and mortality associated with ICM [4].

Current treatment options for ICM and LV functional remodeling primarily focus on relieving symptoms, preventing further myocardial damage, and improving quality of life [3]. These include pharmacological therapies, lifestyle modifications, and invasive procedures, such as percutaneous coronary intervention (PCI) [5]. However, these treatments have limitations, particularly in patients with severe ICM, who are at a higher risk of complications during PCI. Furthermore, some patients may present with cardiogenic shock, refractory ventricular arrhythmias, or mechanical complications that necessitate hemodynamic and/or respiratory support. In such instances, extracorporeal membrane oxygenation (ECMO) can be employed as a bridge to recovery or definitive therapy [6].

ECMO is typically instituted when conventional medical and interventional therapies are insufficient to maintain adequate tissue oxygenation and hemodynamic stability. The decision to initiate ECMO support is based on several factors, including the presence of cardiogenic shock, refractory hypoxemia, or respiratory failure, as well as the patient’s overall clinical condition and prognosis. By providing temporary circulatory and respiratory support, ECMO can potentially improve PCI outcomes by reducing myocardial ischemia during the procedure and allowing for more extensive revascularization in high-risk patients. However, the clinical benefits and impact of ECMO-assisted PCI on LV functional remodeling in ICM patients still need to be fully understood.

This paper presents a single-center retrospective observational study of 76 cases, aiming to increase understanding by analyzing the clinical outcomes and changes in LV functional remodeling in patients with ICM who underwent ECMO-assisted PCI. By providing a comprehensive analysis of these cases, we hope to contribute to the existing body of knowledge and potentially guide future clinical practice in this critical area of cardiology.

## 2. Materials and Methods

### 2.1 Study Design and Population

We conducted a retrospective study of patients with ischemic cardiomyopathy who received ECMO-assisted PCI at our institution from January 2013 to December 2022. The institutional review board (Ethics approval number: 2023ZH135) approved the study protocol, and the need for informed consent was waived due to the retrospective nature of the study. We included patients who (1) were aged 18 years or older, (2) had a left ventricular ejection fraction (LVEF) ≤35% on pre-procedure transthoracic echocardiography (TTE), and (3) had hemodynamic instability requiring ECMO support before or during PCI, including patients with STEMI who were in cardiogenic shock and patients with complex, high-risk anatomy. We excluded patients who (1) had other types of cardiomyopathy (e.g., dilated cardiomyopathy, hypertrophic cardiomyopathy), (2) had a previous cardiac transplantation, and (3) had incomplete medical records.

### 2.2 Data Collection and Outcome Measures

We collected data on demographic characteristics, medical history, laboratory tests, angiographic findings, procedural details, ECMO parameters, and in-hospital complications from the electronic medical records. The SYNTAX score was ascertained using the online SYNTAX score calculator (http://www.syntaxscore.com/calculator/start.htm). The primary outcome was the change in LV functional remodeling from baseline to 12 months post-procedure, measured by transthoracic echocardiography. The secondary outcomes were (1) the incidence of major adverse cardiac and cerebrovascular events (MACCEs), which was a composite outcome of death, reinfarction, cerebrovascular accident, or repeat revascularization. MACCEs were identified using the International Classification of Diseases, Clinical Modification, Tenth Revision (ICD–10-CM) diagnosis codes [7]; (2) the incidence of ECMO-related complications, such as bleeding, infection, limb ischemia, or hemolysis; (3) the length of hospital stay and the intensive care unit stay. Follow-up data were obtained from outpatient visits, telephone interviews, or the national death registry.

### 2.3 ECMO-Assisted PCI Procedure and Postsurgical Treatment 

Our institution is a high-volume tertiary center with extensive experience in advanced heart failure therapies and a dedicated ECMO team comprising cardiovascular surgeons, interventional cardiologists, perfusionists, and intensive care specialists. The decision to utilize ECMO, LVAD, or other therapies, including surgical revascularization (e.g., CABG), is based on a multidisciplinary evaluation of each patient’s clinical status, comorbidities, coronary anatomy, risk profile, and overall prognosis. In patients with ischemic cardiomyopathy, ECMO support was initiated based on hemodynamic instability, cardiogenic shock, or significant left ventricular dysfunction as judged by the treating cardiologist and cardiac surgeon. We excluded patients with severe peripheral vascular disease, active bleeding, or mechanical complications of myocardial infarction. The patients underwent general anesthesia before ECMO implantation. Vascular access, selection of revascularization strategy and devices, use of hemodynamic support devices, and periprocedural pharmacotherapy were at the discretion of the operator.

Venoarterial ECMO was instituted percutaneously or surgically with cannulation of the common femoral vein and artery. Anticoagulation was achieved with heparin to maintain an activated clotting time of 180–220 seconds. The ECMO circuit consisted of a centrifugal pump and hollow-fiber microporous membrane oxygenator. The ECMO flow rate was adjusted to maintain a mean arterial pressure of 60–80 mmHg and a mixed venous oxygen saturation of >70%. Distal limb perfusion was maintained during femoral cannulation. PCI was performed via the femoral or radial artery using contemporary techniques. Thrombus aspiration, predilatation, and drug-eluting stent implantation were performed at the operator’s discretion. An intra-aortic balloon pump was used selectively. The operator determined the PCI strategy to achieve complete revascularization whenever feasible. Completeness of revascularization was defined angiographically as no residual stenosis ≥70% in major epicardial vessels ≥2.5 mm in diameter and all stenotic lesions in branch vessels supplying viable myocardium. ECMO weaning was attempted after hemodynamic stabilization, defined as a cardiac index of >2.2 L/min/m^2^, a central venous pressure of <15 mmHg, and a lactate level of <2 mmol/L. After surgery, patients were admitted to the intensive care unit and received routine postoperative care. Mechanical ventilation was discontinued when patients were normothermic, hemodynamically stable, and awake.

### 2.4 Echocardiography

The echocardiographic measurements were conducted in accordance with the guidelines established by the American Society of Echocardiography and the European Society of Echocardiography [8]. LVEF was measured via the biplane Simpson’s method by combining apical four- and two-chamber views. The wall motion score index (WMSI) was assessed using a 16-segment model of the LV. Each segment with a clear endocardial border was scored as follows: 1 for normal, 2 for hypokinesis, 3 for akinesis, and 4 for dyskinesis. Global WMSI was calculated by summating the scores divided by the number of analyzed segments. Additionally, end-diastolic volume index (EDVI) and end-systolic volume index (ESVI) were calculated using the modified Simpson’s rule and indexed to body surface area.

### 2.5 Medication and Follow-Up

After the ECMO-assisted PCI procedure, patients were prescribed guideline-directed medical therapy (GDMT) for heart failure with reduced ejection fraction, according to the current guidelines from the American College of Cardiology/American Heart Association and the European Society of Cardiology. The GDMT included angiotensin-converting enzyme inhibitors (ACEIs) or angiotensin receptor blockers (ARBs), beta-blockers, mineralocorticoid receptor antagonists (MRAs), and sodium–glucose co-transporter 2 inhibitors (SGLT2i) when indicated. Diuretics were prescribed as needed to manage volume overload. Antiplatelet therapy, statins, and other secondary prevention medications were also prescribed as appropriate.

During the follow-up period, patients were closely monitored in the outpatient clinic, with regular assessments of clinical status, laboratory tests, and echocardiographic evaluations. Medication adjustments were made based on the patient’s response, tolerability, and any adverse effects. Cardiac rehabilitation and lifestyle modifications, including exercise, dietary changes, and smoking cessation, were strongly encouraged.

### 2.6 Statistical Analysis

We expressed continuous variables as mean ± standard deviation or median (interquartile range (IQR)), depending on their distribution, and compared them using Student’s *t*-test or Wilcoxon signed-rank test, respectively. We presented categorical variables as counts or percentages and compared them using the appropriate chi-squared or Fisher’s exact test. A generalized linear mixed-effects model (GLMM) was used to account for repeated measures within patients and to model the longitudinal changes in LVEF, WMSI, EDVI, and ESVI over time. To investigate potential differences in the trends of LV functional remodeling between subgroups, we performed additional GLMM analyses. We compared the LVEF, WMSI, EDVI, and ESVI trends between survivors and non-survivors and between patients who achieved complete revascularization and those who did not. The GLMM included fixed effects for time, subgroup (survivors vs. non-survivors or complete vs. incomplete revascularization), and the interaction between time and subgroup while also accounting for repeated measurements per patient over time. The GLMM for LVED, WMSI, EDVI, and ESVI included fixed effects for time and the aforementioned covariates (clinical indication, baseline values, multivessel disease, collateral circulation, previous MI, postoperative re-infarction, and complete revascularization). These covariates were included as fixed effects in the GLMM to adjust for their potential influence on the LV functional parameters over time. Statistical significance was defined as a two-sided *p*-value of <0.05. The statistical software used throughout the analysis was SPSS v.26.0 (IBM SPSS Inc., Armonk, NY, USA) and R 4.0.1 (R Foundation for Statistical Computing, Vienna, Austria).

## 3. Results

### 3.1 Baseline Characteristics

The study comprised 76 patients with ischemic cardiomyopathy who underwent ECMO-assisted PCI. Table 1 shows the baseline characteristics of the patients. The mean age was 62.67 ± 8.34 years and 66% was male. The majority of patients had a history of hypertension (70%), 33% of the patients had a previous myocardial infarction and 17% had received coronary artery bypass grafting. The mean baseline LVEF was 29.98 ± 2.65%. The majority of patients (n = 68, 89%) presented with cardiogenic shock, while the remaining patients were considered at high risk for anatomy during an elective PCI (n = 3, 4%), or had hemodynamic instability during an elective PCI procedure (n = 5, 7%). Most patients (88%) had a Killip classification of III or IV. Thirteen patients (17%) had previously received coronary artery bypass grafting. The mean SYNTAX score was 29.1 ± 6.4 in patients without previous coronary artery bypass grafting (n = 63).

**Table 1.** **Comparison of baseline demographics and clinical characteristics of survivors and non-survivors**.

Variables ^*^		Total sample (n = 76)	Patient groups		
			Survivors (n = 51)	Non-survivors (n = 25)	*p*-value
Age, years		62.67 ± 8.34	62.11 ± 8.60	63.80 ± 7.84	0.876
Male, n (%)		50 (66%)	33 (65%)	17 (68%)	0.776
BMI, kg/m^2^		25.98 ± 5.27	26.26 ± 5.21	25.40 ± 5.44	0.506
Previous myocardial infarction, n (%)		25 (33%)	13 (25%)	12 (48%)	0.089
Previous PCI, n (%)		15 (20%)	7 (14%)	8 (32%)	0.050
Prior CABG, n (%)		13 (17%)	6 (12%)	7 (28%)	0.149
Comorbidities					
	Hypertension, n (%)	53 (70%)	33 (65%)	20 (80%)	0.173
	History of smoking, n (%)	22 (29%)	16 (31%)	6 (24%)	0.506
	Atrial fibrillation, n (%)	13 (17%)	8 (16%)	5 (20%)	0.885
	COPD, n (%)	10 (13%)	4 (7.8%)	6 (24%)	0.110
	Diabetes mellitus, n (%)	20 (26%)	16 (31%)	4 (16%)	0.153
	Stroke history, n (%)	15 (20%)	8 (16%)	7 (28%)	0.337
	Renal insufficiency/dialysis, n (%)	9 (12%)	5 (9.8%)	4 (16%)	0.683
	Liver dysfunction, n (%)	13 (17%)	8 (16%)	5 (20%)	0.885
	Peripheral artery disease, n (%)	6 (8%)	2 (3.9%)	4 (16%)	0.167
Killip classification III/IV, n (%)		67 (88%)	44 (86%)	23 (92%)	0.728
Clinical indication					0.047
	Stable angina, n (%)	3 (4%)	3 (6%)	0 (0%)	
	Unstable angina, n (%)	37 (49%)	29 (57%)	8 (32%)	
	NSTEMI, n (%)	20 (26%)	9 (18%)	11 (44%)	
	STEMI, n (%)	16 (21%)	10 (20%)	6 (24%)	
MR ≥grade 3, n (%)		15 (20%)	7 (14%)	8 (32%)	0.337
Baseline LVEF, %		29.98 ± 2.65	29.59 ± 2.80	30.76 ± 2.13	0.069

BMI, body mass index; PCI, percutaneous coronary intervention; CABG, coronary artery bypass grafting; COPD, chronic obstructive pulmonary disease; NSTEMI, non–ST-segment elevation myocardial infarction; STEMI, ST-segment elevation myocardial infarction; MR, mitral regurgitation; LVEF, left ventricular ejection fraction. *Continuous data are presented as the mean ± standard deviation, and categorical data are expressed as numbers (%). Non-normally distributed variables are reported as the median (interquartile range (IQR)).

### 3.2 Procedural Details, Clinical Outcome, and Survival Analysis

Table 2 shows the details and outcomes of the ECMO-assisted PCI procedure. Emergent PCI was performed in 62% of patients. The median time of the procedure was 48.14 ± 10.53 minutes. Multivessel disease was present in 82% of the patients, and chronic total occlusion (CTO) was present in 13%. The rates of complete revascularization and CTO treatment were 58% and 29%, respectively. The number of stents implanted was 2 (IQR: 2–3). The median length of stay was 11 days (IQR: 9–13), and the median duration of ECMO support was 38.99 hours (IQR: 26.78–51.2). Most patients (86%) were connected to ECMO before PCI, while 15% were connected during PCI. The most common ECMO-related complications were bleeding (8%) and lower extremity ischemia (5%).

**Table 2.** **Procedure details and in-hospital outcomes of the population (n = 76)**.

Variables ^*^		Total sample (n = 76)	Patient groups		
			Survivors (n = 51)	Non-survivors (n = 25)	*p*-value
Emergent PCI, n (%)		47 (62%)	29 (57%)	18 (72%)	0.202
Time of PCI procedure, mins		46.5 (40–52.25)	47 (40–52)	46 (41–56)	0.965
Angiographic finding in affected coronary arteries					
	Pre-PCI SYNTAX score ^a^	28 (25–35)	27 (24–32)	34 (28.25–37.5)	0.018
	Post-PCI SYNTAX score ^a^	14 (12–17)	14 (12–17)	14.5 (13–16.75)	0.396
	Multivessel disease ^b^, n (%)	62 (82%)	25 (49%)	19 (76%)	0.046
	Patients with CTO, n (%)	10 (13%)	3 (5.9%)	7 (28%)	0.020
	Number of CTO lesions, n	14	5	9	
	Rates of vessels treated, %	76% (137/180)	78% (95/122)	72% (42/58)	0.422
	Collateral circulation, n (%)	16 (21%)	13 (25%)	3 (12%)	0.175
	Rates of CTOs treated, %	29% (4/14)	60% (3/5)	11% (1/9)	0.095
	Complete revascularization, n (%)	44 (58%)	34 (67%)	10 (40%)	0.027
Length of stay (days)		11 (9–13)	11 (8.5–13)	11 (10–13)	0.806
ICU stay (days)		8 (6–11)	7 (5.5–10)	10 (8–12)	0.008
Connection to ECMO before PCI, n (%)		52 (68%)	36 (71%)	16 (64%)	0.561
Connection to ECMO during PCI, n (%)		24 (32%)	15 (29%)	9 (36%)	0.751
Duration of ECMO support (hours)		38.99 ± 26.78	33.71 ± 22.45	49.76 ± 31.78	0.030
Successful mechanical weaning, n (%)		66 (87%)	51 (100%)	15 (60%)	<0.001
Failed ECMO weaning, n (%)		10 (13%)	0 (0%)	10 (40%)	<0.001
Successful ECMO weaning but in-hospital death, %		11% (8/76)	0% (0/51)	32% (8/25)	<0.001
ECMO complications, n (%)					
	Lower extremity ischemia	4 (5%)	1 (2.0%)	3 (12%)	0.195
	Bleeding	6 (8%)	2 (3.9%)	4 (16%)	0.167
	Hemolysis	3 (4%)	1 (2.0%)	2 (8.0%)	0.250
Early clinical outcomes					
	Mortality all cause (hospital), n (%)	18 (24%)	0 (0%)	18 (24%)	<0.001
	Cardiovascular mortality (hospital), n (%)	12 (16%)	0 (0%)	12 (16%)	<0.001
	Intra-aortic balloon pump support, n (%)	40 (53%)	25 (49%)	15 (60%)	0.368
	Re-infarction (hospital)	5 (7%)	1 (2.0%)	4 (16%)	0.068
	Neurological complications, n (%)	13 (17%)	6 (11.8%)	7 (28%)	0.149
	CRRT, n (%)	8 (11%)	3 (12%)	5 (9.8%)	>0.999
	Blood transfusion, n (%)	63 (83%)	38 (75%)	25 (100%)	0.014
	Respiratory failure, n (%)	40 (53%)	24 (47%)	16 (64%)	0.165
Late clinical outcome					
	One-year mortality, n (%)	23 (30%)	0 (0%)	23 (92%)	<0.001

Abbreviations: PCI, percutaneous coronary intervention; CTO, chronic total occlusion; ICU, intensive care unit; ECMO, extracorporeal membrane oxygenation; CRRT, continuous renal replacement therapy. ^*^ Continuous data are presented as the mean ± standard deviation, and categorical data are expressed as numbers (%). Non-normally distributed variables are reported as the median (interquartile range (IQR)). ^a^ The SYNTAX score was computed exclusively for patients who had not undergone prior CABG—63 patients. ^b^ Multivessel coronary artery disease (MVD) is characterized by a luminal stenosis of 70% or more in a minimum of two major coronary arteries or one coronary artery coupled with a stenosis of the left main trunk exceeding 50%.

**Fig. 1.**
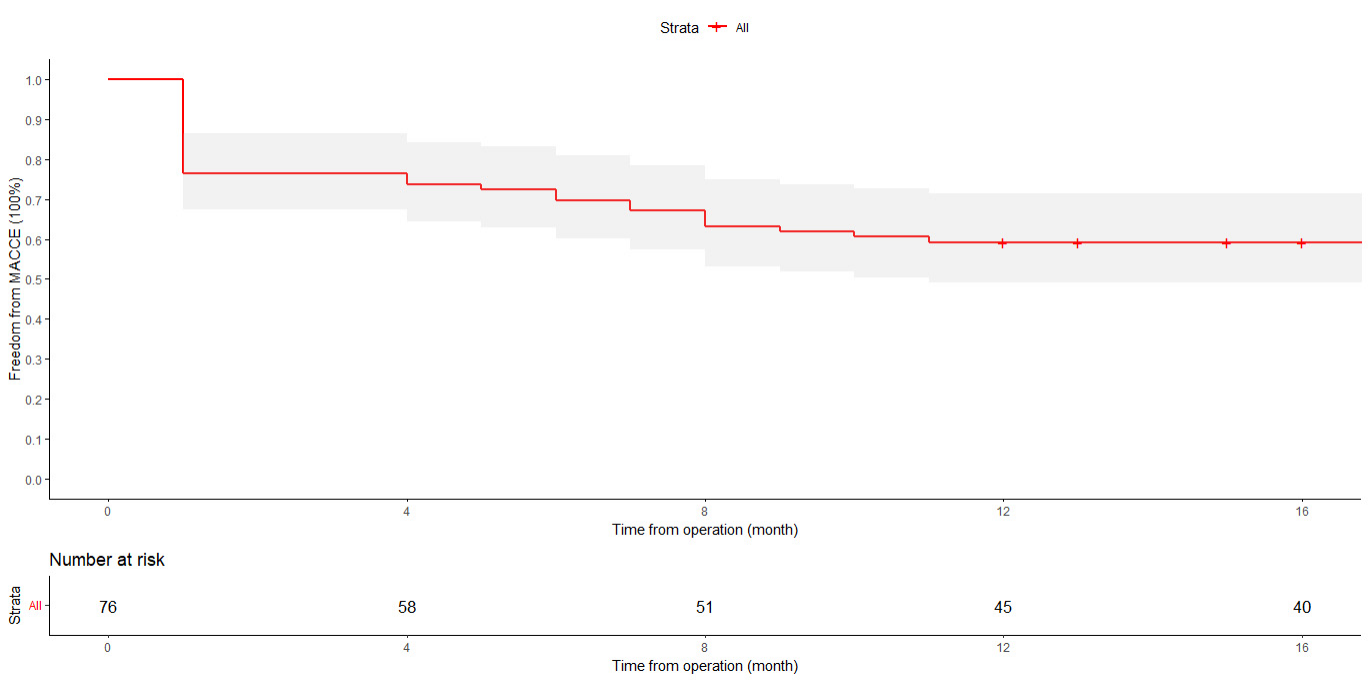
**Kaplan–Meier curve showing MACCE-free survival with a standard error of <10% during or before the 16-month point**. MACCE, major adverse cardiac and cerebrovascular event.

The Kaplan–Meier curve for freedom from MACCEs is shown in Fig. 1. The overall freedom from MACCEs at 12 months was 59% (95% confidence interval (CI): 49–71%). In the subgroup with complete revascularization, the freedom from MACCEs at 12 months was 73% (95% CI: 61–87%). Conversely, in the subgroup with incomplete revascularization, the freedom from MACCEs at 12 months was 41% (95% CI: 27–62%). The difference between these two subgroups was statistically significant (log-rank test: *p*-value = 0.018). The most common MACCE within 12 months was death (30%), followed by cerebrovascular accident (21%), reinfarction (20%), and repeat revascularization (5%). The one-year mortality rate was 30%. The observed in-hospital mortality rate was 24%, with the cardiovascular mortality rate being 18%. Cardiovascular mortality was attributed to various causes, including cardiac arrest (two cases), refractory cardiogenic shock (five cases), ventricular arrhythmia (three cases), and other cardiovascular-related causes (four cases). The overall survival rate after 12 months was recorded at 70%, while the cardiovascular-specific survival rate in the same period was 82%.

### 3.3 Medication Use

Before the ECMO-assisted PCI procedure, 60 patients (78%) were receiving inotropic support, including dobutamine, dopamine, or milrinone. Vasodilators, such as nitrates or sodium nitroprusside, were administered to 40 patients (53%). Other vasoactive medications, such as norepinephrine or vasopressin, were used in 28 patients (37%) to maintain adequate perfusion pressure. At the 12-month follow-up, among the 53 surviving patients, 50 patients (94%) were on aspirin, 53 patients (100%) were on a P2Y12 inhibitor (clopidogrel), 42 patients (79%) were on beta-blockers, 49 patients (92%) were on an angiotensin-converting enzyme inhibitor (ACEI) or angiotensin receptor blocker (ARB), 53 patients (100%) were on a statin, and 53 patients (100%) were on a mineralocorticoid receptor antagonist (MRA). These medications were prescribed in accordance with the current guidelines for the management of heart failure and coronary artery disease.

### 3.4 Changes in Left Ventricular Functional Remodeling 

**Fig. 2.**
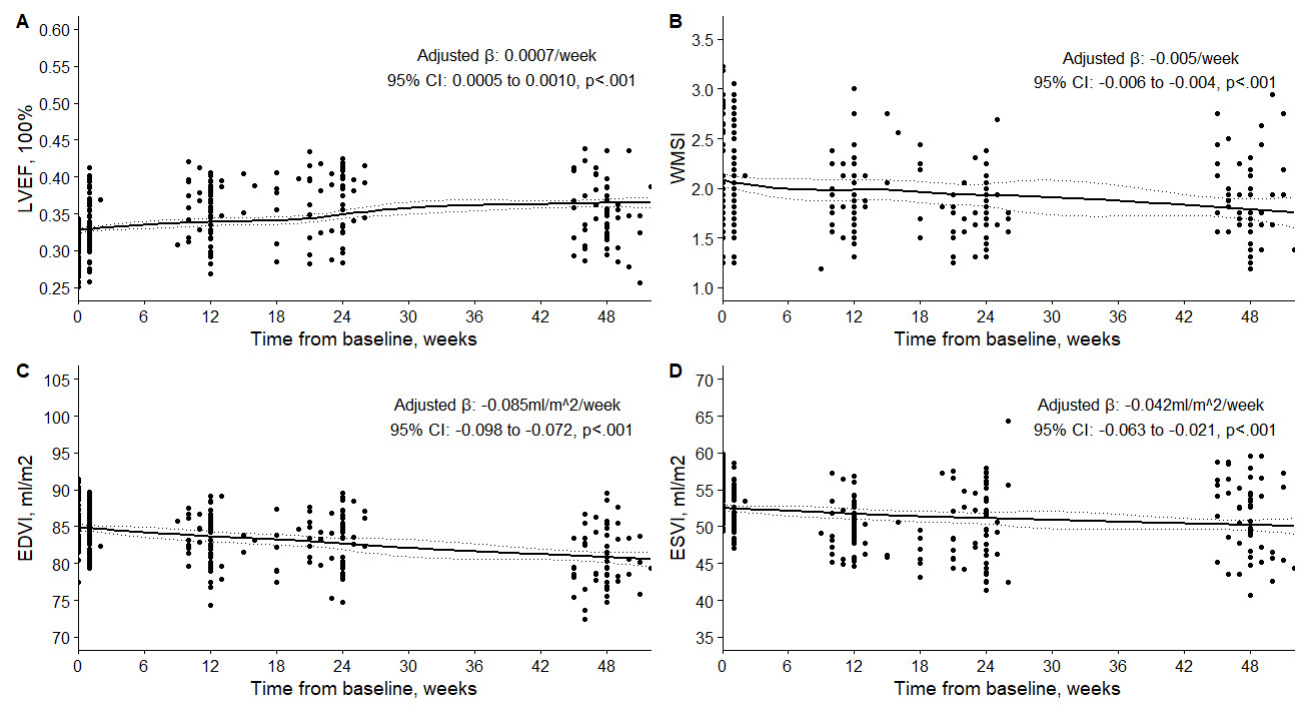
**Changes in LVEF (A), WMSI (B), EDVI (C), and ESVI (D) in the patients at baseline and follow-up**. LVEF, left ventricular ejection fraction; WMSI, wall motion score index; EDVI, end-diastolic volume index; ESVI, end-systolic volume index.

The changes in LV functional remodeling from baseline to 12 months after the procedure are shown in Table 3 and Fig. 2. A GLMM was used to account for repeated measures within patients and to model the longitudinal changes in these variables over time. There was a significant improvement in LVEF, WMSI, EDVI, and ESVI over time (*p* < 0.001 for all) after adjusting for clinical indication, baseline values of LVEF, WMSI, EDVI, and ESVI, as well as multiple vessel disease, collateral circulation, previous myocardial infarction, postoperative re-infarction, and complete revascularization. The results showed that LVEF increased significantly after the procedure from baseline to 6 months, with a weekly change rate of 0.0007/week (95% CI: 0.0005 to 0.0010/week; *p* < 0.001). However, LVEF decreased slightly at 1 year, although it was still higher than the baseline value. WMSI, EDVI, and ESVI decreased significantly from baseline to 1 year, indicating improved LV function and reduced LV size. The weekly change rates for WMSI, EDVI, and ESVI were –0.005/week (95% CI: –0.006 to 0.004/week), –0.085 mL/m^2^/week (95% CI: –0.098 to 0.072 mL/m^2^/week), and –0.042 mL/m^2^/week (95% CI: –0.063 to 0.021 mL/m^2^/week), respectively (*p* < 0.001 for all). These results suggest that ECMO-assisted PCI positively impacted LV functional remodeling in patients with ICM.

**Table 3.** **GLMM for changes in LV functional remodeling pre- and postoperatively**.

Variables ^a^	Baseline	1 week	3 month	6 month	1 year	β ^b^	*p*-value
	(n = 76)	(n = 68)	(n = 56)	(n = 56)	(n = 54)		
LVEF ^c^	29.98 ± 2.65	34.33 ± 3.71	35.73 ± 3.72	36.41 ± 4.33	35.16 ± 4.32	0.0007	<0.001
WMSI ^c^	85.36 ± 3.01	84.51 ± 2.92	83.17 ± 3.43	83.16 ± 3.63	80.80 ± 3.91	–0.005	<0.001
EDVI ^c^	54.32 ± 3.22	52.08 ± 2.78	49.81 ± 3.26	49.69 ± 5.26	51.69 ± 5.06	–0.085	<0.001
ESVI ^c^	53.95 (51.45–57.55)	51.55 (50.175–54.6)	49.45 (47.775–51.8)	48.65 (45.475–53.975)	52.75 (47.35–55.525)	–0.042	<0.001

Abbreviations: GLMM, generalized linear mixed-effects model; LVEF, left ventricular ejection fraction; EDVI, end-diastolic volume index; ESVI, end-systolic volume index; WMSI, wall motion score index; LV, left ventricular. ^a^ Continuous data are presented as the mean ± standard deviation. Non-normally distributed variables are reported as the median (interquartile range (IQR)). ^b^ β is the weekly change rate of LVEF, EDVI, ESVI, WMSI, in percentage/week, mL/m^2^/week, mL/m^2^/week, no unit/week, respectively, in a generalized linear mixed-effects model after adjusting for clinical indication, baseline values of LVEF, WMSI, EDVI, and ESVI, as well as multiple vessel disease, collateral circulation, previous myocardial infarction, postoperative re-infarction, and complete revascularization. ^c^ LVEF is in percentage; EDVI and ESVI are in mL/m^2^; WMSI has no unit.

**Fig. 3.**
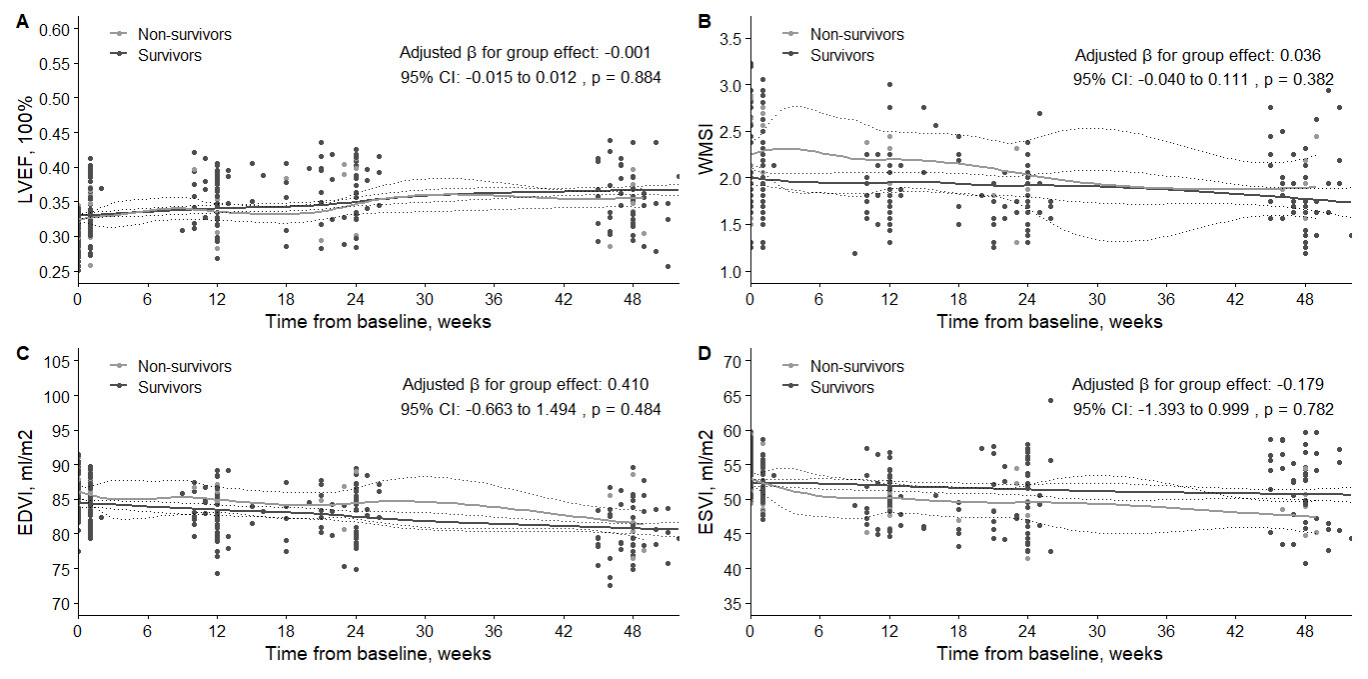
**Comparison of changes in LVEF (A), WMSI (B), EDVI (C), and ESVI (D) between survivor and non-survivor patient groups at baseline and follow-up**. LVEF, left ventricular ejection fraction; WMSI, wall motion score index; EDVI, end-diastolic volume index; ESVI, end-systolic volume index.

**Fig. 4.**
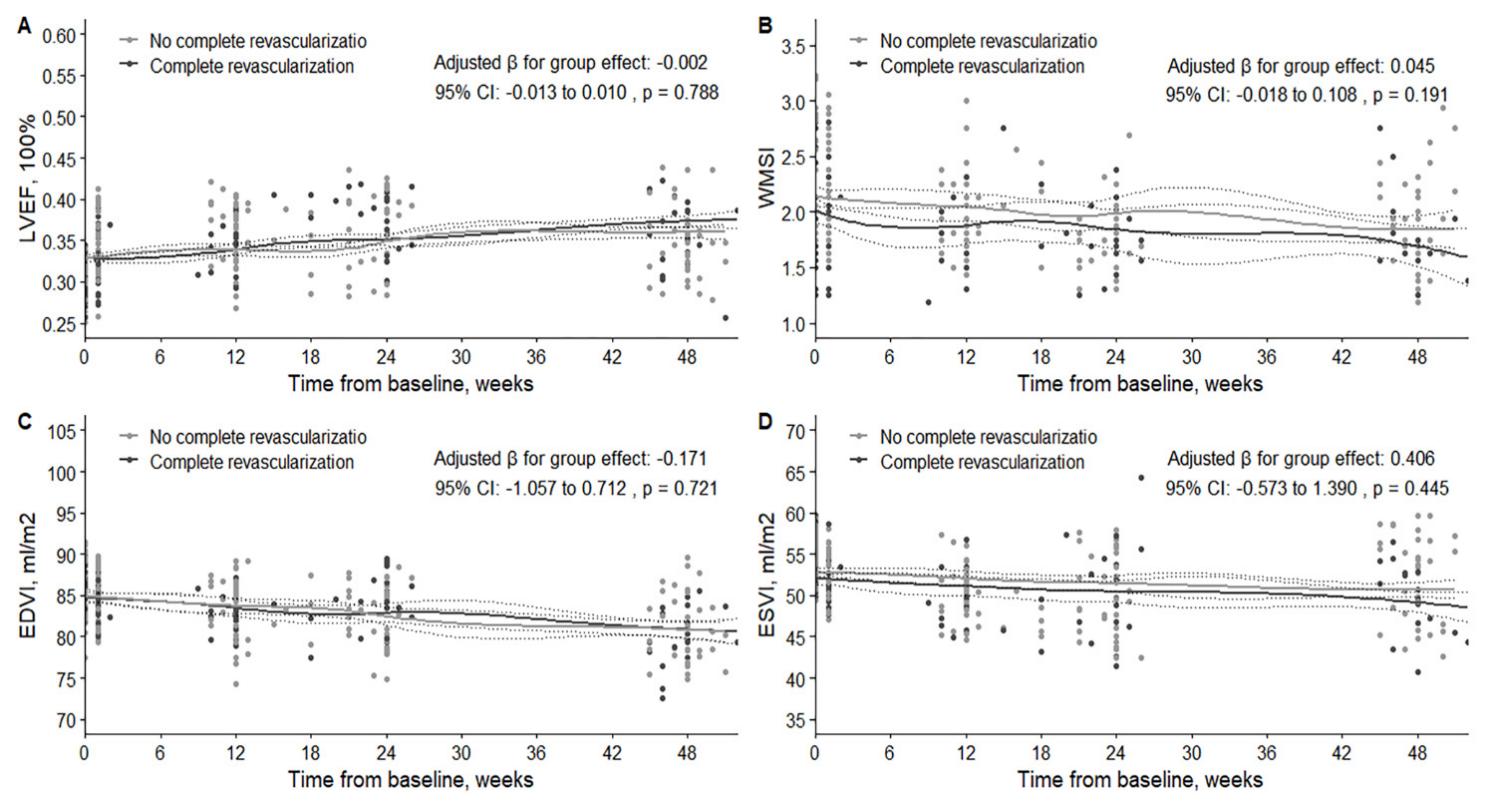
**Comparison of changes in LVEF (A), WMSI (B), EDVI (C), and ESVI (D) between patients with incomplete and complete revascularization at baseline and follow-up**. LVEF, left ventricular ejection fraction; WMSI, wall motion score index; EDVI, end-diastolic volume index; ESVI, end-systolic volume index.

The GLMM analysis did not reveal a significant difference between survivors and non-survivors in LVEF, WMSI, EDVI, and ESVI improvement trends over time. The adjusted β for the group effect was –0.001 (95% CI: –0.015 to 0.012, *p* = 0.884) for LVEF; 0.036 (95% CI: –0.040 to 0.111, *p* = 0.382) for WMSI; 0.410 (95% CI: –0.663 to 1.494, *p* = 0.484) for EDVI; –0.179 (95% CI: –1.393 to 0.999, *p* = 0.782) for ESVI. Furthermore, the analysis did not demonstrate a significant difference in LVEF, WMSI, EDVI, and ESVI improvement between the subgroups with incomplete and complete revascularizations. The adjusted β for the group effect was –0.002 (95% CI: –0.013 to 0.010, *p* = 0.788) for LVEF; 0.045 (95% CI: –0.018 to 0.108, *p* = 0.191) for WMSI; –0.171 (95% CI: –1.057 to 0.712, *p* = 0.721) for EDVI; 0.406 (95% CI: –0.573 to 1.390, *p* = 0.445) for ESVI. These findings are illustrated in Figs. 3,4.

## 4. Discussion

The present study aimed to investigate the impact of ECMO-assisted PCI on clinical outcomes and LV functional remodeling in patients with ICM. The main findings of this study are as follows: (1) ECMO-assisted PCI was feasible and safe in patients with ICM, with a high rate of complete revascularization and a low rate of ECMO-related complications; (2) ECMO-assisted PCI was associated with a significant improvement in LV functional remodeling, as evidenced by the increase in LVEF and the decrease in WMSI, EDVI, and ESVI at 12 months follow-up; (3) ECMO-assisted PCI was associated with a favorable survival rate at 12 months, despite the high-risk profile of the patients.

The effect of ECMO-assisted PCI on left ventricular remodeling in ICM patients has yet to be extensively investigated. While previous studies have reported the feasibility and safety of ECMO-assisted PCI in patients with cardiogenic shock or cardiac arrest due to acute myocardial infarction [9, 10], there is limited research focused on ICM patients who exhibit chronic and progressive deterioration of LV function. Furthermore, most previous studies have primarily assessed short-term clinical outcomes, such as in-hospital mortality or 30-day mortality, and have not evaluated long-term changes in LV function or remodeling [11, 12]. Therefore, our study provides novel and valuable insights into the potential benefits of ECMO-assisted PCI in patients with ICM.

In patients with ICM, reduced LV function and reserve, multivessel disease, chronic total occlusions, or left main lesions may present a high risk of hemodynamic collapse during PCI [13, 14], particularly if the procedure is prolonged or complicated. ECMO has the potential to address these challenges by providing temporary hemodynamic stabilization, reducing myocardial ischemia, and facilitating complete revascularization. In our study, we achieved a high rate of complete revascularization (58%) and CTO treatment (29%) in patients with ICM, which may have contributed to the improvement in LV functional remodeling and survival [15].

The remarkable improvement in LV functional remodeling after ECMO-assisted PCI in our study is noteworthy, considering the baseline characteristics of the patients. The mean baseline LVEF was 29.98%, indicating severe impairment of LV function. The majority of the patients were in cardiogenic shock, which is known to be associated with worse LV function and prognosis. Additionally, the mean SYNTAX score was 29.1, reflecting the high complexity and extent of the coronary disease. Despite these unfavorable factors, we observed a significant increase in LVEF and a notable decrease in WMSI, EDVI, and ESVI at the 12-month follow-up, indicating an enhancement in LV systolic function and a reduction in LV size. These changes in LV functional remodeling may have resulted from the reduction in myocardial ischemia and infarct size, the prevention of adverse LV remodeling, and the enhancement in myocardial viability and contractility after ECMO-assisted PCI. These findings are consistent with previous studies that reported improved LV function following ECMO support in patients with cardiogenic shock [12, 16]. The possible mechanisms of ECMO-assisted PCI on LV functional remodeling include (1) reduction in myocardial ischemia and infarct size via provision of adequate coronary perfusion and oxygen delivery by ECMO [17]; (2) unloading of the LV through lowered LV afterload, subsequently reducing LV end-diastolic volume [18]; (3) facilitation of complete revascularization by ECMO, which can enhance myocardial viability and contractility. Our study utilized a GLMM to examine the longitudinal alterations in LV functional remodeling over time. A notable strength of the GLMM is its capacity to manage missing data, a particularly relevant consideration for our study due to patient deaths during the follow-up period.

Expanding on this, the utilization of GDMT in our patient cohort aimed to impede the progression of heart failure, diminish hospital readmissions, and enhance survival rates. We closely monitored patients to optimize GDMT, making necessary adjustments based on their response to therapy, renal function, blood pressure, and other clinical parameters. Equally significant, reinforcing adherence to GDMT was prioritized during follow-up visits, and patients were educated on the pivotal role of these medications in managing their condition. This approach ensures a comprehensive treatment strategy that supplements the mechanical support ECMO provides.

It is important to note that our study was conducted in a high-volume tertiary center with extensive experience in advanced heart failure therapies and a dedicated ECMO team. The feasibility and safety of ECMO-assisted PCI observed in our study may not be generalizable to centers with less experience or resources. Furthermore, the rate of complete revascularization in our study was modest at 58%, which may have influenced the clinical outcomes. Future studies should investigate strategies to improve the completeness of revascularization in this challenging patient population.

The 12-month survival rate following ECMO-assisted PCI in our study was promising, considering the high-risk profile of the patients. The one-year mortality rate was 30%, with an in-hospital mortality rate of 24%. These rates either align with or are lower than the reported mortality rates of ECMO-assisted PCI in previous studies [19, 20]. The in-hospital cardiovascular mortality rate was 18%. These findings indicate that ECMO-assisted PCI may improve the survival of patients with ICM by providing effective circulatory support and enabling complete revascularization. However, it is important to note that other factors, including patient selection, the indication, and timing of ECMO initiation, the duration and mode of ECMO support, postoperative management, and follow-up care, may have influenced the survival rate in our study. Therefore, cautious interpretation and comparison of survival outcomes of ECMO-assisted PCI across different studies are warranted.

The incidence of ECMO-related complications in our study was low, underscoring the safety and feasibility of ECMO-assisted PCI in patients with ICM and hemodynamic instability. The most common ECMO-related complications were bleeding (8%) and lower extremity ischemia (5%), consistent with the reported rates of these complications in prior studies [21]. Notably, no instances of infection, hemolysis, or stroke—rare but serious complications of ECMO—were observed. The diminished frequency of ECMO-related complications in our study may be credited to the careful patient selection, meticulous ECMO implantation and management, the implementation of distal limb perfusion, and the timely ECMO weaning and decannulation.

The study results revealed a significant association between complete revascularization and higher freedom rates from MACCEs, underscoring the importance of complete revascularization in improving patient prognosis. Notably, subgroup analyses demonstrated that improvements in LV functional remodeling parameters were not significantly different between the compared subgroups (survivors vs. non-survivors and incomplete vs. complete revascularization). However, the lack of statistical significance does not necessarily imply the absence of clinically relevant differences between the subgroups. Future research should explore strategies to optimize patient selection for ECMO-assisted PCI further and assess the impact of this intervention on various subgroups of patients with ICM, utilizing larger sample sizes and longer follow-up periods to understand better the factors influencing LV functional remodeling in patients with ischemic cardiomyopathy undergoing ECMO-assisted percutaneous coronary intervention.

The study’s limitations primarily stem from its retrospective and observational design, potentially leading to selection bias, confounding factors, and missing data. The relatively small sample size and short follow-up period could have restricted the statistical power and the generalizability of the results. Additionally, the lack of blinding in the echocardiographic measurements performed by various operators may have introduced interobserver and intraobserver variability. The discretionary use of ECMO and PCI by operators may have resulted in heterogeneity in the indication, timing, and technique of these interventions. Consequently, further prospective randomized controlled trials are needed to confirm the efficacy and safety of ECMO-assisted PCI in patients with ICM.

## 5. Conclusions

In conclusion, our single-center retrospective study suggests that ECMO-assisted PCI may be a feasible and safe therapeutic option for patients with ischemic cardiomyopathy and hemodynamic instability when performed in a high-volume tertiary center with a dedicated ECMO team. However, the modest rate of complete revascularization highlights the need for further research to optimize the procedural outcomes. Despite these limitations, ECMO-assisted PCI was associated with a significant improvement in LV functional remodeling and a favorable 12-month survival rate in this high-risk patient population. Prospective, multicenter studies are warranted to validate these findings and to refine the patient selection and device management strategies for ECMO-assisted PCI.

## Availability of Data and Materials

All data generated or analyzed during this study are included in this published article.

## Abbreviations

ICM, ischemic cardiomyopathy; LV, left ventricular; ECMO, extracorporeal membrane oxygenation; PCI, percutaneous coronary intervention; LVEF, left ventricular ejection fraction; MACCEs, major adverse cardiac and cerebrovascular events; WMSI, wall motion score index; EDVI, end-diastolic volume index; ESVI, end-systolic volume index; GDMT, guideline-directed medical therapy; GLMM, generalized linear mixed-effects model; IQR, interquartile range; CTO, chronic total occlusion; CI, confidence interval.
